# FUS interacts with nuclear matrix-associated protein SAFB1 as well as Matrin3 to regulate splicing and ligand-mediated transcription

**DOI:** 10.1038/srep35195

**Published:** 2016-10-12

**Authors:** Atsushi Yamaguchi, Keisuke Takanashi

**Affiliations:** 1 Department of Neurobiology, Graduate School of Medicine, Chiba University, Chiba, Japan

## Abstract

*FUS (Fused-in-Sarcoma)* is a multifunctional DNA/RNA binding protein linked to familial amyotrophic lateral sclerosis/frontotemporal dementia (ALS/FTD). Since FUS is localized mainly in the nucleus with nucleo-cytoplasmic shuttling, it is critical to understand physiological functions in the nucleus to clarify pathogenesis. Here we report a yeast two-hybrid screening identified FUS interaction with nuclear matrix-associated protein SAFB1 (scaffold attachment factor B1). FUS and SAFB1, abundant in chromatin-bound fraction, interact in a DNA-dependent manner. N-terminal SAP domain of SAFB1, a DNA-binding motif, was required for its localization to chromatin-bound fraction and splicing regulation. In addition, depletion of SAFB1 reduced FUS’s localization to chromatin-bound fraction and splicing activity, suggesting SAFB1 could tether FUS to chromatin compartment thorough N-terminal DNA-binding motif. FUS and SAFB1 also interact with Androgen Receptor (AR) regulating ligand-dependent transcription. Moreover, FUS interacts with another nuclear matrix-associated protein Matrin3, which is muted in a subset of familial ALS cases and reportedly interacts with TDP-43. Interestingly, ectopic ALS-linked FUS mutant sequestered endogenous Matrin3 and SAFB1 in the cytoplasmic aggregates. These findings indicate SAFB1 could be a FUS’s functional platform in chromatin compartment to regulate RNA splicing and ligand-dependent transcription and shed light on the etiological significance of nuclear matrix-associated proteins in ALS pathogenesis.

*FUS (Fused-in-sarcoma*), one of causative genes for familial amyotrophic lateral sclerosis/frontotemporal dementia (ALS/FTD), encodes a multifunctional DNA/RNA binding protein. FUS is a nucleo-cytoplasmic shuttling protein with multiple domains consisting of N-terminal Gln-Gly-Ser-Tyr (QGSY) rich region, Glycine (G)-rich region, RNA recognition motif (RRM), two Arg-Gly-Gly (RGG)-rich repeats, zinc finger motif, and C-terminal nuclear localization signal (NLS). Various mutations in FUS-related familial ALS/FTD patients are clustered in C-terminal NLS, leading to cytoplasmic mislocalization and inclusion^1^,^2^,^3^,^4^. N-terminal QGSY and G-rich region is low-complexity (LC) domain that is implicated in prion-like polymerization to form nonmembranous compartments such as RNA granules^5^,^6^. Although the etiological significance of cytoplasmic inclusion remains elusive, FUS localizes mainly in the nucleus where it plays a variety of roles including transcriptional regulation, RNA metabolism (splicing, transport, translation, and degradation), and DNA damage repair^1^,^2^,^3^,^4^. In addition, FUS is associated with active chromatin and interacts with various nuclear proteins, including nuclear matrix, C-terminal domain of RNA polymerase II, a subset of splicing and transcriptional factors^7^,^8^,^9^,^10^,^11^.

Chromatin is thought to be organized in ordered structures consisting of radial looped domains fixed to the nuclear matrix/scaffold, which is a proteinaceous fibrogranular framework resistant to high salt and detergent extraction. The nuclear matrix/scaffold is thought to consist of two main structures; ‘internal nuclear matrix’ and ‘nuclear shell’ (nuclear lamina). Nuclear matrix-associated proteins (nuclear matrix proteins), bounding to the internal nuclear matrix/scaffold, are thought to form a skeletal nuclear framework with roles in chromatin organization, DNA replication, DNA repair, transcriptional regulation, and RNA metabolism. The regions of the genome that bind to the nuclear matrix/scaffold were named as scaffold/matrix attachment regions (S/MARs), which are usually AT-rich sequences. The interaction of the genome within this nuclear framework could act coordinately with epigenetic modifications to modulate chromatin permissive or silencing states^12^,^13^,^14^. Scaffold attachment factor B1 (SAFB1), considered as a nuclear matrix-associated protein, was originally identified by independent two groups, based on its ability to bind to S/MAR^15^ or the promoter of hsp27^16^. SAFB1 is a large multi-domain protein characterized by the N-terminal SAP domain (or SAF box) critical for binding to S/MARs and contains RRM, NLS, Q/R-rich, and G-rich domain. SAFB1 interacts with RNA polymerase II, a subset of splicing factors (SR proteins and hnRNPs), and chromatin remodeling factor CHD (chromodomain helicase DNA-binding) and is involved in multiple functions including chromatin remodeling, DNA damage response, and RNA metabolism. SAFB1 is one of SAFB protein family consisting of SAFB1, SAFB2, and SATB1 with sharing 74% amino acid similarity between SAFB1 and SAFB2. SAFB family may be part of a ‘transcriptosome’ complex coupling transcriptional regulation and RNA processing^17^,^18^,^19^,^20^,^21^.

Matrin3, considered as a component of nuclear matrix-associated complex, is a DNA/RNA binding protein with multi-domains, containing NLS and NES (nuclear export signal), two zinc finger domains and two RRM, which can anchor chromosomes to the nucleus matrix thorough binding to the S/MAR. The mutations in Matrin3 are linked with distal myopathy and ALS, while Matrin3 interact with ALS/FTD-associated TDP-43^22^,^23^,^24^,^25^.

In the present study, we identified SAFB1 as a novel interacting partner of FUS through a yeast two-hybrid screening on human brain cDNA library. We also found FUS interacts with Matrin3 in co-immunoprecipitation assay. We then examined the functional significance of the interplay between FUS and SAFB1 in mammalian cultured cells.

## Results

### SAFB1 interacts with FUS tethering it to chromatin-bound fraction

**Figure 1:**
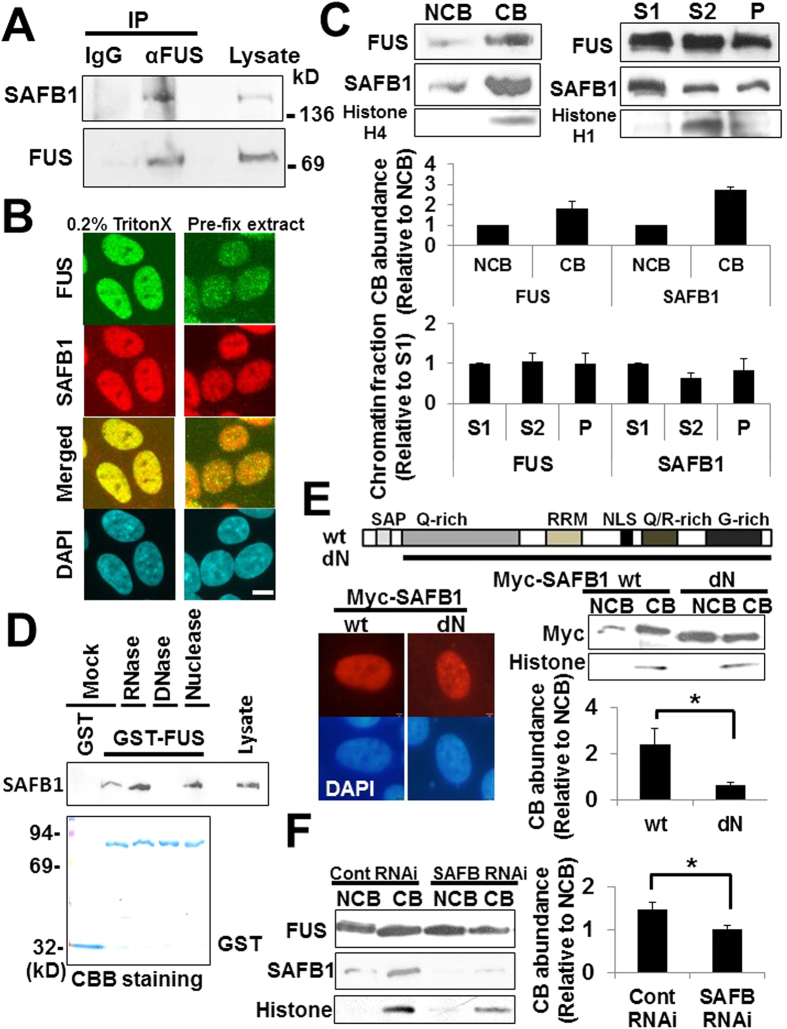
SAFB1 interacts with FUS tethering it to chromatin-bound fraction. (**A**) Co-immunoprecipitation assay between FUS and SAFB1. HEK293 lysates after sonications were immunoprecipitated with IgG (control) or anti-FUS antibody, followed by immunoblot with anti-SAFB1 (upper panel) and anti-FUS antibody (lower panel). (**B**) SH-SY5Y cells were fixed (4% PFA), and permeabilized (0.2% TritonX-100) [Left panels] or treated with CSK buffer with 0.5% TritonX-100 as described in ‘Materials and Methods’ [Right panels]. Then cells were co-stained with anti-FUS (#sc-47711) and -SAFB antibody followed by fluorescent secondary antibodies. Images were obtained with confocal microscopy after DAPI staining. Bars, 5 μm. (**C**) Chromatin-bound (CB) fraction, non-chromatin-bound (NCB) fraction, and Chromatin subfractions (S1,S2,P) were prepared in HEK293. The abundance of each fraction was shown as ratio relative to NCB or S1 fraction in graphs. The data are presented as the mean values ± SD of a total of three times. (**D**) GST (control) and GST-FUS protein were incubated with lysates of HEK293 over-expressing myc-tagged SAFB1. Then samples were treated with RNase, DNase I or micrococcal nuclease. After washing, samples were subjected to Western blot with anti-SAFB1 antibody (upper panel). The lower panel shows the Coomassie brilliant blue (CBB)-stained SDS-polyacrylamide gel with GST-fused proteins. (**E**) The diagrams indicate the structure of SAFB1 wt and dN. [Left panels] HEK293 cells transfected with myc-tagged SAFB1 wt or dN plasmid were stained with anti-myc antibody followed by fluorescent secondary antibody. Images were obtained with fluorescence microscopy after DAPI staining. Bars, 2 μm. [Right panels] Distribution pattern of myc-SAFB1 wt and dN in the chromatin fraction. HEK293 cells, transfected with myc-SAFB1 wt or dN plasmid, were processed for chromatin fraction assay. The abundance of CB fraction was shown as ratio relative to NCB fraction in graphs. The data are presented as the mean values ± SD of a total of three times. *p < 0.05 with Student’s *t*-test. (**F**) Distribution pattern of FUS in the chromatin fraction with siRNA-mediated SAFB knockdown. HEK293 with siRNA-mediated SAFB knockdown were processed for chromatin fraction assay. The data are presented as the mean values ± SD of a total of three times. *p < 0.05 with Student’s *t*-test.

To get insights into the physiological functions of FUS, we performed a yeast two-hybrid screen on human brain cDNA library to identify binding partners of FUS as described previously^26^. One of positive clones was cDNA encoding full-length *Scaffold Attachment Factor B 1 (SAFB1*) (NCBI; XM_006722839). First to confirm the interaction, we performed co-immunoprecipitation (Co-IP) assay in HEK293 cells. Since SAFB1 is thought to be nuclear matrix-associated protein resistant to high salt and detergent extraction, we performed Co-IP assay after shearing chromatin with sonications as previously reported^27^. Cell lysates were immunoprecipitated with anti-FUS antibody, and subjected to Western blot with anti-SAFB1 antibody (Fig. 1A). SAFB1 protein was present in the immunoprecipitate with anti-FUS antibody, indicating the interaction between FUS and SAFB1 in sheared chromatins. Next to investigate the subcellular localization, we conducted immunocytochemistry in neuronal SH-SY5Y cells. The distribution pattern of FUS in the nucleus was different dependent on the fixation, permeabilization method, and antibody (Supplementary Fig. 1A,B). FUS distributed diffusely in the extra-nucleolar region of the nucleus with 4% paraformaldehyde fixation and 0.2% Triton X-100 permeabilization, whereas it showed a punctate or granular pattern in the nucleus with methanol fixation or pre-fix extraction with detergent (0.5% Triton X-100) in CSK buffer as previously reported^28^,^29^. FUS and SAFB1 were observed diffusely throughout the nucleoplasm with various intensities in 4% paraformaldehyde fixation and 0.2% Triton X-100 permeabilization (Fig. 1B, left panels), while FUS showed a punctate pattern with pre-fix extraction by 0.5% Triton X-100 remarkably as compared with SAFB1 (Fig. 1B, right panels) suggesting at least they are not completely co-localized in the nucleus.

Previous reports showed a significant portion of FUS exists in active chromatin fraction^8^,^9^. We then examined whether SAFB1 exists in the active chromatin fraction in HEK293 cells. The chromatin-bound (CB) fraction was obtained after shearing chromatin with sonications as described^8^,^9^. Histone H4 was used as a marker of CB fraction. SAFB1 was present abundantly in CB fraction as well as FUS. The relative ratio of CB to Non-chromatin bound (NCB) fraction of SAFB1 (mean; 2.7 ± 0.15) is higher than that of FUS (mean; 1.9 ± 0.3) (Fig. 1C, left panels and upper graph). To further examine the distribution pattern of SAFB1 in chromatin fractions, cells were processed according to standard protocol^8^,^9^,^30^. Three sequential fractions were obtained: S1 (chromatin soluble in divalent ions, depleted of Histone H1), S2 (EDTA soluble, more compact chromatin), and P (active chromatin and nuclear matrix components) as described^30^. Histone H1 (a protein associated with inactive chromatin) was used as a marker of S2 compact chromatin fraction. The relative ratio of P fraction to S1 fraction of SAFB1 (mean; 0.52 ± 0.32) was comparable to that of FUS (mean; 0.50 ± 0.26) (Fig. 1C, right panels and lower graph).

A variety of RNA-binding proteins, including FUS and TDP43, are assembled in ribonucleoprotein (RNP) complex in an RNA-dependent manner. The interaction between FUS and SAFB1 remained in Co-IP assay with RNase treatment (Supplementary Fig. 2A). Then to address whether FUS interacts with SAFB1 in a nucleotide-dependent manner, we performed pull down assay with the treatment of DNaseI, Micrococcal nuclease or RNase. Micrococcal nuclease digestion is commonly used to allow for double-strand breaks within nucleosome linker regions^31^. Bacterially expressed GST-fused FUS (GST-FUS) was incubated overnight with lysate of HEK293 over-expressing SAFB1 protein after shearing chromatin with sonications. Samples were then treated with mock, RNase, DNase I, or Micrococcal nuclease followed by the immunoblot with anti-SAFB1 antibody (Fig. 1D). The band showing the interaction between GST-FUS and SAFB1 were present in the treatment with mock, RNase, or Micrococcal nuclease but not with DNase I, suggesting FUS could interact with SAFB1 in a DNA-dependent manner in this pull down assay.

As in Fig. 1E, SAFB1 contains N-terminal SAP domain (also known as SAF-box), which is involved in binding to AT-rich S/MARs and is often found in proteins to regulate higher order chromatin structure^18^,^19^,^20^. To address whether SAP domain is critical for SAFB1 to localize to CB fraction, we prepared the construct encoding N-terminal deletion mutant SAFB1 dN (Fig. 1E). Morphologically the subnuclear localization of SAFB1 dN appeared similar to that of SAFB1 wild type (wt) (Fig. 1E, left panels). In contrast, the relative ratio of CB to NCB fraction of SAFB1 dN (mean; 0.6 ± 0.10) was significantly lower than that of SAFB1 wt (mean; 2.4 ± 0.68) (Fig. 1E, right panels and graph).

Then to investigate the effect of SAFB1 on FUS’s localization to chromatin fraction, we conducted chromatin fraction assay with small interfering RNA (siRNA)-mediated SAFB knockdown. Since SAFB1 and SAFB2 share 74% similarity at amino acid level, mixture of SAFB1- and SAFB2-specific siRNA was used in the treatment of siRNA-mediated SAFB knockdown (Supplementary Fig. 2B) as previously described^21^. The relative ratio of CB to NCB fraction of FUS was reduced in the treatment with siRNA-mediated SAFB knockdown (mean; 1.0 ± 0.08) compared with that of control siRNA (mean; 1.5 ± 0.18) (Fig. 1F).

Then to examine which domain of FUS is required for binding to SAFB1, we performed pull-down assay using GST-fused FUS deletion mutants (GST-FUS-NT and GST-FUS-CT) (Supplementary Fig. 2D). The binding was observed only in the reaction using GST-FUS wt (Supplementary Fig. 2D), indicating full-length of FUS might be required for the binding in this assay.

### Morphological characterization of FUS and SAFB1 in spinal cord and in SH-SY5Y

**Figure 2:**
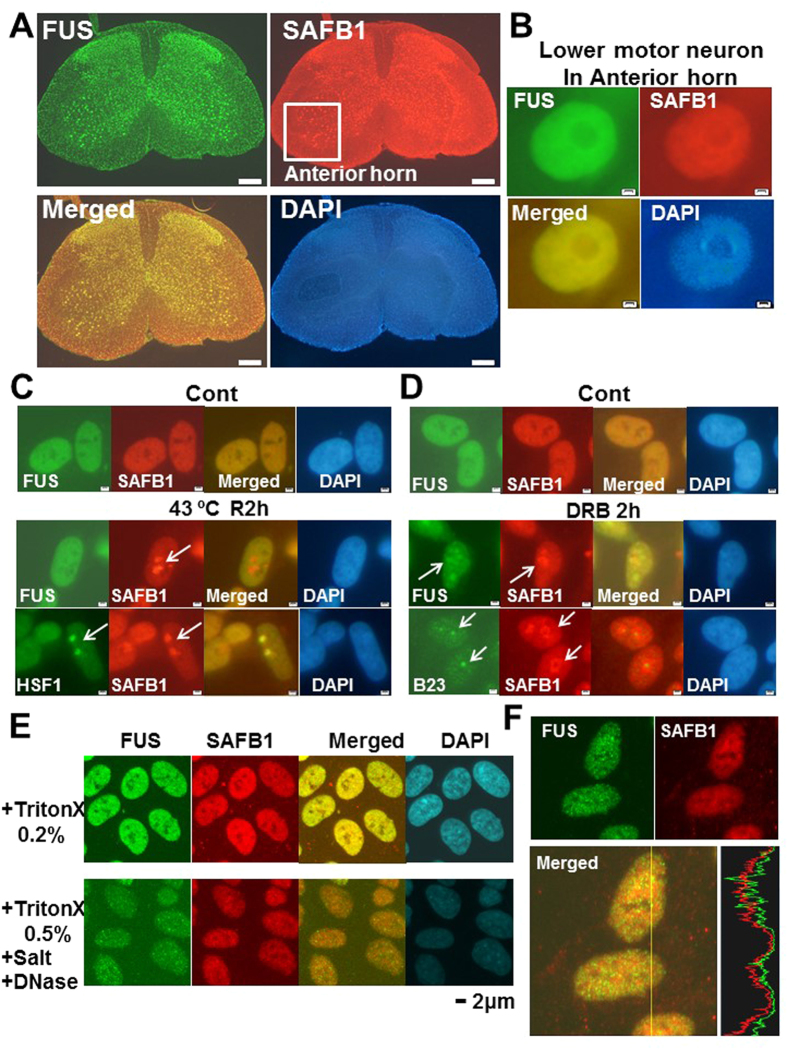
Morphological characterization of FUS and SAFB1 in spinal cord and in SH-SY5Y. (**A**) Lumbar spinal cord sections of 6 weeks old male mouse were co-stained with anti-FUS(#sc-47711) and anti-SAFB antibody followed by fluorescent secondary antibodies. Images were obtained with fluorescence microscope after DAPI staining. Bars, 200 μm. (**B**) Magnified images of lower motor neurons in the anterior horn of spinal cord. Bars; 2 μm. (**C**) SH-SY5Y cells were exposed to heat stress (43 °C, 60 min), and then returned to 37 °C for 2 h. Cells were fixed (4% PFA), permeabilized (0.2% TritonX), and co-stained with anti-FUS (#sc-47711) and -SAFB1 antibody (top and middle panels) or anti-HSF1 and -SAFB1 antibody (bottom panels), followed by fluorescent secondary antibodies. Images were obtained under fluorescence microscope after DAPI staining. Arrows show the localizations for indicated protein. Bars, 2 μm. (**D**) SH-SY5Y cells, treated with 40 μg/ml DRB for 2 h, were fixed (4% PFA), permeabilized (0.2% TritonX), and co-stained with anti-FUS (#sc-47711) and -SAFB antibody (top and middle panels) or anti-B23 and -SAFB1 antibody (bottom panels), followed by fluorescent secondary antibodies. Bars, 2 μm. (**E**) *In situ* nuclear matrices in SH-SY5Y were prepared as described in ‘Materials and Methods’. Upper panels (+TritonX 0.2%) are control images treated only with 0.2% TritonX-100. Lower panels (+TritonX 0.5%, +Salt, +DNase) are images of *in situ* nuclear matrices. Images co-stained with anti-FUS (#sc-47711) and -SAFB antibody were obtained with confocal microscope (Olympus FV10) after DAPI staining. (**F**) The intensity profile of the fluorescence signals along with the line was shown on the right merged panel.

SAFB1 is expressed in most tissues with very high expression in brain^18^,^19^,^20^. To examine whether SAFB1 is expressed in motor neurons, we performed immunhistochemistry on mouse spinal cord sections. FUS and SAFB1 were both observed diffusely throughout the nucleoplasm in lower motor neurons, which harbor typically large cell bodies and are localized in the anterior horn of spinal cord (Fig. 2A,B).

Then we investigated the subnuclear localization of FUS and SAFB1 in neuronal SH-SY5Y cells. SAFB1 relocates into nuclear granules called as nuclear Stress Body (nSB) in response to heat shock stress, which was originally identified as the main site of heat-shock factor-1 (HSF1) accumulation in stressed cells^32^. To address whether FUS is also recruited to nSB upon heat shock stress, we performed immunocytochemistry in SH-SY5Y exposed to heat shock (43 °C, 1 h) followed by recovery incubation (37 °C, 2 h). SAFB1 relocated to nSB that is stained with anti-HSF1 antibody at 2 h after heat shock (Fig. 2C, bottom panels), whereas FUS was not recruited there in that condition (Fig. 2C, middle panels). On the other hand, FUS is recruited to perinucleolar region, called as ‘perinucleolar cap’ with the treatment of RNA polymerase inhibitors including 5,6-dichloro-1-beta-D-ribofuranosylbenzimidazole (DRB) and Actinomycin D^28^. We then examined whether SAFB1 relocates to perinucleolar region with DRB treatment. When SH-SY5Y cells were treated with DRB for 2 h, both FUS and SAFB1 were recruited to the region around nucleolus that was stained with anti-B23 (nucleophosmin) antibody (Fig. 2D, bottom panels). FUS and SAFB1 were partially co-localized around the nucleolus (Fig. 2D, middle panels).

FUS is also present in the nuclear matrices^7^. We then investigated the distribution pattern of FUS and SAFB1 in the nuclear matrices morphologically by *in situ* nuclear matrix assay. SH-SY5Y cells on cover slips were treated with non-ionic detergents, high-salt buffer, and nuclease (‘+TritonX 0.5%, +Salt, + DNase’ in Fig. 2E) to obtain nuclear matrices as described^33^, while control sample was treated with only 0.2% TritonX-100 (‘+Triton X 0.2%’ in Fig. 2E). FUS in the nucleus was distributed in a punctate pattern as described^7^, while SAFB1 was observed diffusely in the nucleus (Fig. 2E). Speckles of FUS *in situ* nuclear matrix were partially co-localized with those of speckles stained with anti-SC35 antibody (Supplementary Fig. 3). In the intensity profile of the fluorescence signals, the intensity peaks of FUS were partially merged with those of SAFB1 (Fig. 2F).

### Cooperative interplay between SAFB1 and FUS in alternative splicing

**Figure 3:**
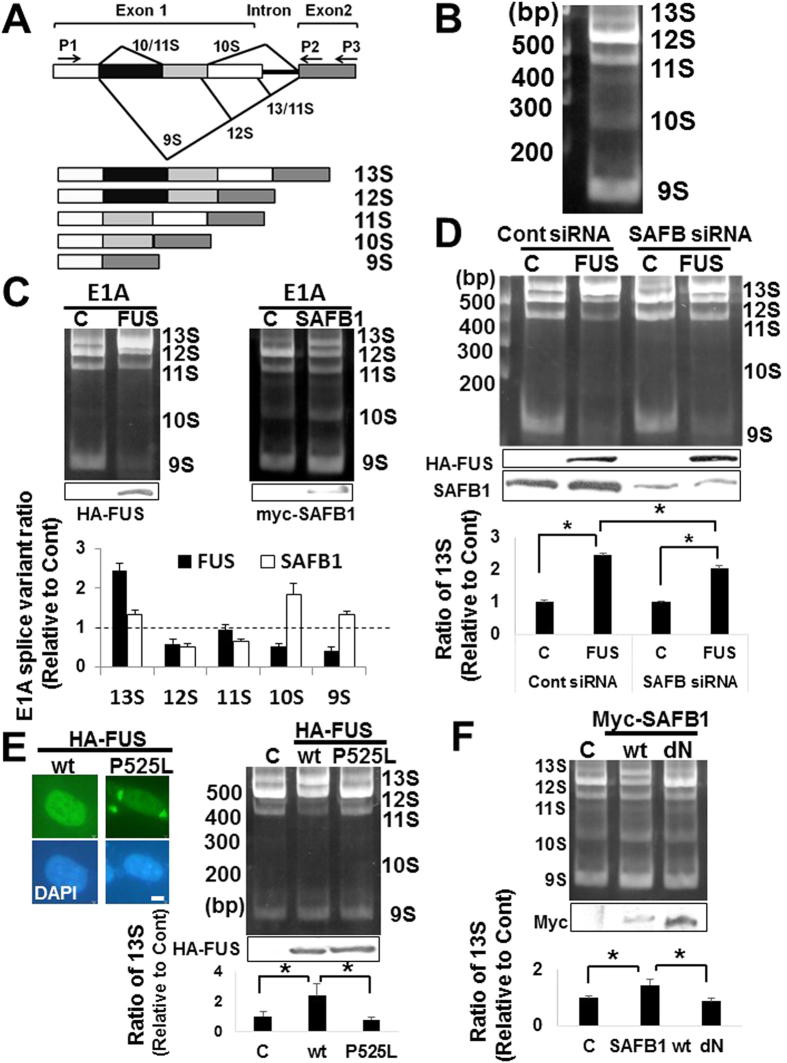
Effects of FUS and SAFB1 on *in vivo* alternative pre-mRNA splicing. (**A**) The diagrams show the alternative splicing pattern of the E1A mini-gene. The arrows (P1,P2) indicate the binding sites of primers for RT-PCR, and the arrow (P3) indicates that of primer for reverse-transcription. (**B**) Representative image of major splicing products of E1A mini-gene in HEK293. (**C**) HEK293 cells were co-transfected with the E1A reporter plasmid and HA-FUS (left panel) or myc-SAFB1 vector (right panel) as indicated. Empty vector was used as control. At 48 h post-transfection, cells were processed for splicing assay. The abundance of the major splicing products was shown as ratio relative to control in the lower graph. The data are presented as the mean values ± SD that were repeated a total of three times. (**D**) HEK293 cells were co-transfected with the E1A reporter plasmid and HA-FUS vector at 12 h after siRNA-mediated knockdown of SAFB. Total RNAs were isolated 48 h post-transfection, and processed for splicing assay. The abundance of the 13S splicing variant was shown as ratio relative to control in the lower graph. The data are presented as the mean values ± SD that were repeated a total of three times. *p < 0.05 by two-way ANOVA with Bonferroni post-hoc tests. (**E**) [Left panels] HEK293 cells were transfected with HA-FUS wt or P525L plasmid and stained with anti-HA antibody followed by fluorescent secondary antibody. Images were obtained with fluorescence microscope after DAPI staining. [Right panels] HEK293 cells were co-transfected with the E1A reporter plasmid and control, HA-FUS wt, or HA-FUS P525L construct. At 48 h post-transfection, cells were processed for splicing assay. The data are presented as the mean values ± SD that were repeated a total of three times. *p < 0.05 by one-way ANOVA with Bonferroni post-hoc tests. (**F**) HEK293 cells were co-transfected with the E1A reporter plasmid and control, myc-SAFB1 wt or dN construct. At 48 h post-transfection, cells were processed for splicing assay. The data are presented as the mean values ± SD that were repeated a total of three times. *p < 0.05 by one-way ANOVA with Bonferroni post-hoc tests.

Over-expression of FUS or SAFB1 modulates the splicing patterns of adenovirus-derived E1A mini-gene^17^,^34^. To investigate the interplay between FUS and SAFB1 in the alternative splicing, we performed splicing assays using E1A mini-gene construct in HEK293. HEK293 cells were transiently transfected with E1A mini-gene plasmid, and RNA products amplified by RT (reverse transcription)-PCR were separated by gel electrophoresis. In the basal conditions, the transcripts of E1A mini-gene were alternatively spliced to 5 isoforms (13S, 12S, 11S, 10S, 9S) (Fig. 3A,B) as previously described^17^,^34^. Then we investigated effects of FUS or SAFB1 over-expression on splicing regulation of E1A mini-gene. Over-expression of FUS promoted the splicing of 13S transcript, while that of SAFB1 increased 9S, 10S, and 13S transcript (Fig. 3C). Next to address whether FUS and SAFB1 coordinately regulate pre-mRNA splicing, we performed E1A mini-gene splicing assays with siRNA-mediated SAFB knockdown. The relative levels of 13S transcript were reduced in combination of ectopic FUS and siRNA-mediated SAFB knockdown (mean; 2.02 ± 0.11) compared with that of ectopic FUS and control siRNA (mean; 2.44 ± 0.04) (Fig. 3D).

Then to investigate effects of ALS-linked FUS mutant on the splicing regulation, we performed splicing assays using C-terminal mutant FUS P525L that leads to severe ALS phenotype^1^,^2^,^3^,^4^. Morphologically FUS wild type (wt) localized exclusively in the nucleus, while FUS P525L was mislocalized in the cytoplasm and formed cytoplasmic aggregates in HEK293 (Fig. 3E, left panels) as reported previously^35^. The relative levels of 13S transcript were not increased by FUS P525L over-expression (mean; 0.76 ± 0.16) compared with control in contrast with those of FUS wt (mean; 2.39 ± 0.78) (Fig. 3E, right panels). Then to examine whether N-terminal SAP domain of SAFB1 (Fig. 1E) is critical for splicing function, we performed splicing assays when HEK293 cells were co-transfected with E1A mini-gene and SAFB1 dN construct (Fig. 3F). The relative levels of 13S variant were not increased by SAFB1 dN over-expression (mean; 0.87 ± 0.11) compared with control in contrast with those of SAFB1 wt (mean; 1.45 ± 0.20), suggesting N-terminal SAP domain might be critical for the splicing activity.

### FUS and SAFB1 could coordinately repress AR-mediated transcriptional activity

**Figure 4:**
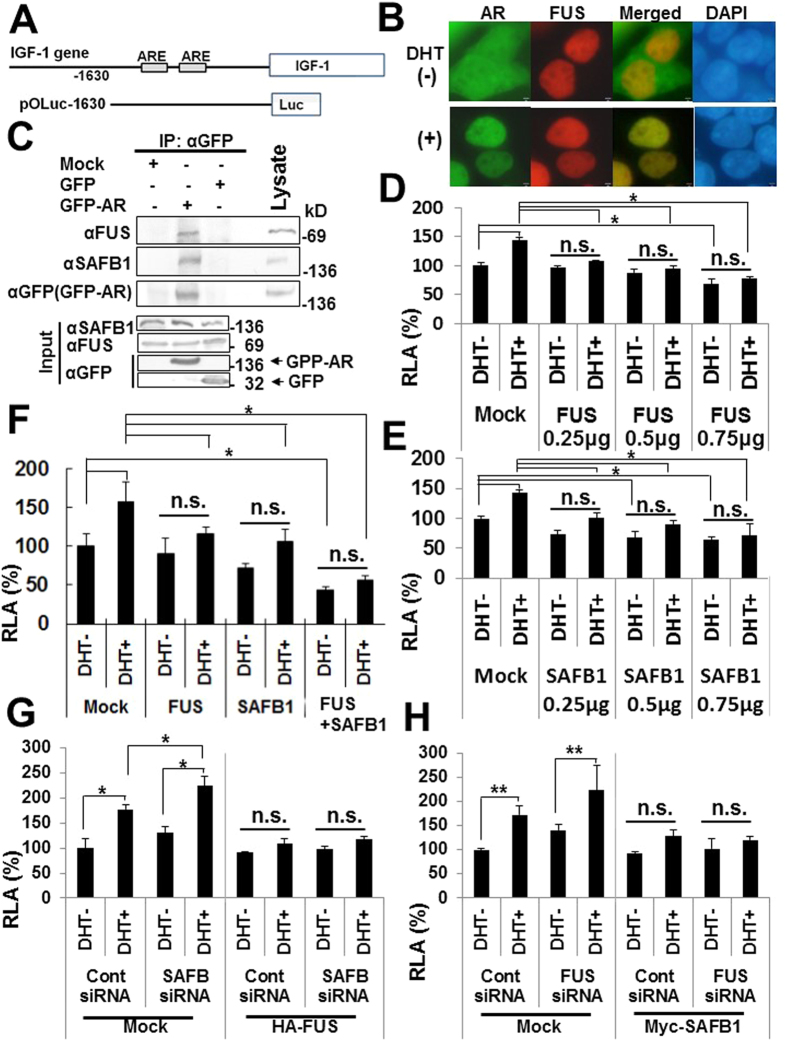
Effects of FUS and SAFB1 on AR-mediated transcription in reporter gene assay. (**A**) The diagrams show the map of androgen-responsive region (ARE) shown as rectangles in upstream promoter of IGF-I gene and pOLuc1630 vector. (**B**) HEK293 cells were transfected with GFP-fused AR (GFP-AR) plasmid for 24 h, and treated with mock (−) or 80 nM DHT (+) for 24 h followed by anti-FUS antibody staininig. Bars, 2 μm. DHT, dihydrotestosterone. (**C**) Lysates of HEK293 over-expressing Mock, GFP-AR or GFP were immunoprecipitated with anti-GFP antibody and then immunoblotted with anti-FUS (upper), anti-SAFB1 (middle), or anti-GFP antibody (lower panel). Immunoblots of input were shown below. (**D,E**) Dose-dependent effects of FUS or SAFB1 on AR-mediated transcription. HEK293 cells were co-transfected with 0.25 μg each of pOLuc1630, GFP-AR, pSV-β-Galactosidase, and 0.25, 0.5, 0.75 μg of FUS or SAFB1 plasmid per well in 24 well plate. Amount of plasmid was equaled with empty vector in each sample. Then cells were treated with Mock (DHT−) or 80 nM DHT (DHT+) for 24 h and sampled for assays. RLA (%), relative luciferase activity. The data are presented as the mean values ± SD of triplicate samples. *p < 0.05 by two-way ANOVA with Bonferroni post-hoc tests. n. s., not significant. (**F**) HEK293 cells were co-transfected with 0.25 g/well each of pOLuc1630, GFP-AR, pSV-β-Galactosidase, and HA-FUS and/or myc-SAFB1 plasmid. Amounts of plasmids were equaled with empty vector in each sample. At 24 h post-transfection, cells were treated with Mock (DHT−) or 80 nM DHT (DHT+) for 24 h. The data are presented as the mean values ± SD of triplicate samples. *p < 0.05 by two-way ANOVA with Bonferroni post-hoc tests. (**G,H**) HEK293 cells, after siRNA-mediated knockdown for 24 h, were co-transfected with ach 0.25 g/well each of pOLuc1630, GFP-AR, pSV-β-Galactosidase, and empty vector (Mock) or HA-FUS/Myc-SAFB1 plasmid. Amounts of plasmids were equaled with empty vector in each sample. At 24 h post-transfection, cells were treated with mock (DHT−) or 80 nM DHT (DHT+) for 24 h. The data are presented as the mean values ± SD of triplicate samples. *p < 0.05, **p < 0.05 by two-way ANOVA with Bonferroni post-hoc tests.

SAFB1 was intensively studies as a co-repressor of estrogen receptor^18^,^19^,^20^, while previous reports showed that FUS and SAFB1 interacts with androgen receptor (AR) to regulate its transcriptional activity, respectively^36^,^37^. Then to investigate the interplay between FUS and SAFB1 on the hormone-mediated transcription, we conducted reporter gene assay using AR-responsive reporter construct. Since IGF-1 promoter contains two possible AR responsive elements (AREs) that harbor induction by androgen (Fig. 4A), we used the reporter construct pOLuc-1630 that contains two AREs of IGF-1 promoter inducible by androgen^38^,^39^.

First to address whether GFP-fused AR (GFP-AR) is responsive to androgen, HEK293 cells transfected with GFP-AR plasmid were treated with 5a-dihydrotestosterone (DHT), an active derivative of testosterone, for 24 h. GFP-AR was diffusely distributed in the cell in basal conditions, whereas it relocated into the nucleus upon DHT treatment morphologically (Fig. 4B). Next to examine whether FUS and SAFB1 interact with AR, we performed Co-IP assay. Lysates of HEK293 over-expressing Mock, GFP-AR or GFP with sonication-sheared chromatin were immunoprecipitated with anti-GFP antibody followed by Western blot with anti-FUS and anti-SAFB1 antibody, respectively. Both FUS and SAFB1 were detected in the immunoprecipitate of GFP-AR expressing cells with anti-GFP antibody (Fig. 4C). We then examined the interplay between FUS and SAFB1 in the regulation of AR-mediated transcription in reporter gene assay. HEK293 cells were co-transfected with pOc1630 Luc and FUS or SAFB1 plasmid, and then treated with DHT for 24 h. Ectopic expression of FUS and SAFB1 repressed AR-mediated transcription in a dose dependent manner, respectively (Fig. 4D,E). Moreover, co-expression of FUS and SAFB1 additively repressed AR-mediated transcription (Fig. 4F). To corroborate these findings with siRNA-mediated knockdown approach (Supplementary Fig. 1B,C), we measured AR-mediated luciferase activities with combination of over-expression and siRNA-mediated knockdown of FUS or SAFB1. SiRNA-mediated knockdown of SAFB abolished the repressive activities of endogenous SAFB on AR-mediated transcription (Fig. 4G, left 4 lanes), which was in part canceled by HA-FUS over-expression (Fig. 4G, right 4 lanes). In the same manner, siRNA-mediated knockdown of FUS abolished the repressive activities of endogenous FUS (Fig. 4H, left 4 lanes), which was in part canceled by Myc-SAFB1 over-expression (Fig. 4H, right 4 lanes). Taken together these results suggest FUS and SAFB1 could regulate AR-mediated transcriptional activities in an additive manner.

### Matrin3 interacts with FUS and is sequestered in the aggregates of ALS-linked FUS mutant

**Figure 5:**
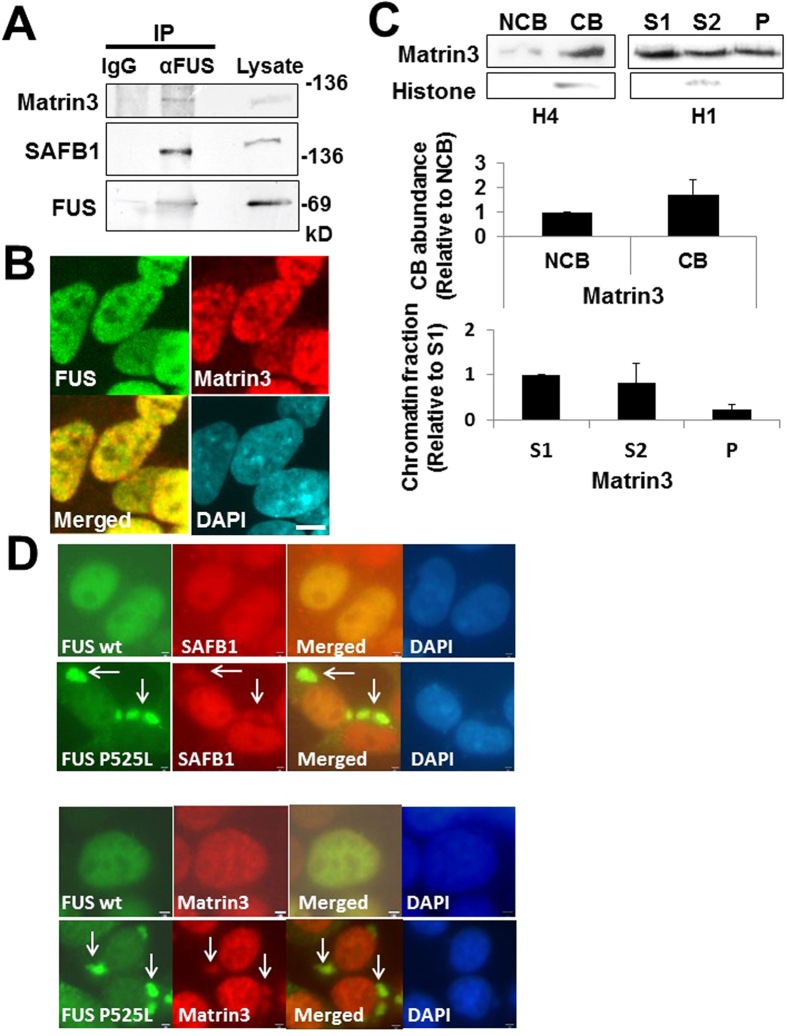
Matrin3 interacts with FUS and is sequestered in the aggregates of ALS-linked FUS mutant. (**A**) Co-IP assay among FUS, Matrin3, and SAFB1. Lysates of HEK293 with sonication-sheared chromatin were immunoprecipitated with mouse IgG (control) or anti-FUS antibody and followed by Western blot with anti-Matrin3 antibody (top panel). The membrane was reprobed with anti-SAFB1 (middle) and -FUS antibody (lower panel). (**B**) SH-SY5Y cells were fixed (4% PFA), permeabilized (0.2% TritonX) and co-stained with anti-FUS (#sc-47711) and –Matrin3 antibody followed by the incubation with fluorescent secondary antibodies. Images were obtained using confocal microscope after DAPI staining. Bars, 5 μm. (**C**) Distribution pattern of Matrin3 in the chromatin fraction. Chromatin-bound (CB) fraction, non-chromatin-bound (NCB) fraction, and Chromatin subfractions (S1,S2,P) were prepared in HEK293. The abundance of each chromatin fraction was shown as ratio relative to NCB or S1 fraction in the lower graphs. The data are presented as the mean values ± SD that were repeated a total of three times. (**D**) HEK293 cells were transfected with HA-FUS wt or P525L plasmid for 48 h. Cells were fixed (4% PFA), permeabilized (0.2% TritonX), and then co-stained with anti-HA and -SAFB1 (upper panels) or -Matrin3 (lower panels) antibody followed by fluorescent secondary antibodies. Images were obtained with fluorescence microscope after DAPI staining. Arrows indicate the co-localizations. Bars, 2 μm.

Matrin3, a DNA/RNA binding protein with multi-domains, is considered as a nuclear matrix-associated protein and linked with ALS. Since previous report showed that Matrin3 interacts with SAFB1 and SAFB2^23^, we then addressed whether FUS interacts with Matrin3 in Co-IP assay. HEK293 lysates with sonication-sheared chromatin were immunoprecipitated with anti-FUS antibody and subjected to Western blot with anti-Matrin3 and -SAFB1 antibody respectively (Fig. 5A). Matrin3 protein as well as SAFB1 was present in the immunoprecipitate with anti-FUS antibody, indicating the interaction among FUS, Matrin3, and SAFB1 in sonication-sheared chromatin fraction.

Next to investigate the subcellular localization of FUS and Matrin3, we conducted immunocytochemistry in SH-SY5Y cells. Both FUS and Matrin3 were observed diffusely throughout the nucleoplasm with various intensities (Fig. 5B). Then we examined the distribution patterns of Matrin3 in chromatin fractions in HEK293 cells. Matrin3 was abundant in CB fraction (Fig. 5C), however the relative ratio of Matrin3 in P fraction to S1 fraction (mean; 0.22 ± 0.12) was relatively low compared with that of FUS and SAFB1 (mean; 0.50 ± 0.26, 0.52 ± 0.32, respectively in Fig. 1C). Since P fraction is thought to be transcriptionally active domains along with subsequent RNA metabolism^8^,^9^,^30^, this might suggest that relatively larger portion of FUS and SAFB1 in chromatin fractions could involve in transcriptional regulation and multiple steps of RNA metabolism.

As shown in Fig. 3E, ectopic FUS P525L is mislocalized in the cytoplasm and forms cytoplasmic aggregates in HEK293. To address whether FUS P525L over-expression sequesters Matrin3 or SAFB1 in the aggregates, we performed immunocytochemistry in HEK293 transfected with HA-FUS P525L plasmid. At 48 h post-transfection, endogenous SAFB1 (upper panels) and Matrin3 (lower panels) were sequestered in the cytoplasmic aggregation of ectopic FUS P525L respectively (Fig. 5D). It was shown the level of FUS in active chromatin fraction is significantly reduced for ALS-linked FUS mutants^8^. We then asked whether FUS P525L over-expression reduces the relative level of SAFB1 in the active chromatin fraction (P) (Supplementary Fig. 4A,B). Consistent with the previous report^8^, the relative level of ectopic HA-FUS P525L in active chromatin fraction was reduced compared with that of HA-FUS wt. However, the relative level of SAFB1 in P fraction was not significantly changed by HA-FUS P525L over-expression (Supplementary Fig. 4B), which might be because the endogenous FUS could remain enough in chromatin fractions even after HA-FUS P525L over-expression.

## Discussion

Nuclear matrix-associated proteins, including SAFB1 and Matrin3, are thought to form a skeletal nuclear framework with roles in chromatin organization, DNA replication, DNA repair, transcriptional regulation, and RNA metabolism^13^,^14^,^16^,^17^,^18^,^19^,^20^,^21^,^23^,^24^,^25^. Most of these functions are similar to those of ALS-linked RNA binding proteins (TDP-43, FUS, and hnRNP A1/A2)^3^,^4^. In fact, SAFB1 was originally identified as a binding partner of hnRNP A1 and once named as HAP (hnRNP A1 associated protein)^18^,^19^,^20^. Matrin3 is muted in a subset of ALS patients and interacts with SAFB1 and TDP-43^23^,^25^. In the present study, we identified SAFB1 as a novel interacting partner of FUS in yeast two-hybrid screening. They were present abundantly in chromatin-bound fraction biochemically and in lower motor neurons of mouse spinal cord morphologically. Their binding was confirmed in Co-IP assay after sonication-mediated chromatin shearing. The interaction disappeared with DNase I treatment in pull down assay, suggesting they interact in the chromatin compartment in a DNA-dependent manner. They coordinately regulated the alternative splicing and repressed AR -mediated transcription *in vitro*. In addition, N-terminal SAP domain of SAFB1 was critical to localize in chromatin-bound fraction and required for the splicing regulation. SAP domain is a non-sequence-specific DNA-binding region that has preference for AT-rich scaffold/matrix attachment regions (S/MARs)^18^,^19^,^20^,^21^, and siRNA-mediated knockdown of SAFB1 reduced the amount of FUS in chromatin-bound fraction. These findings suggest SAFB1 could be a functional platform to tether FUS through N-terminal SAP domain to chromatin compartment where they coordinately regulate splicing and ligand-dependent transcription. Previous studies showed FUS and SAFB1 are associated with polycomb repressive complex 2 (PRC2) that is histone methyltransferase required for epigenetic silencing respectively^37^,^40^, indicating their interaction also could involve in epigenetic gene regulation.

Splicing can occur co-transcriptionally. The modulation of the RNA binding proteins to interact with RNA polymerase II (RNAP II) and the elongation rate of transcription are critical in splicing decisions. Especially carboxy-terminal domain (CTD) of RNAP II has a key role in functionally coupling transcription and splicing^41^. Since FUS and SAFB1 interact with RNAP II CTD and RNA processing proteins^3^,^10^,^18^,^19^,^20^,^21^, their interaction could play a role in coupling transcription and splicing. On the other hand, splice site selection is governed by an intricate balance of various factors^41^, therefore ectopic expression of FUS and/or SAFB1 could affect the splice sites by changing the balance in the present study.

Morphologically the subcellular localization of SAP domain-deleted SAFB1 (SAFB1 dN) was similar to that of wild type in cultured cells, which might be consistent with that SAP domain is not necessary for proper nuclear localization in *Drosophila* larval neuroblast nuclei^42^. Both FUS and SAFB1 relocated to the perinucleolar region in the treatment with RNA polymerase inhibitor, whereas only SAFB1 was recruited into nuclear Stress Body (nSB) along with HSF1 after heat shock stress suggesting their functional differences in stress condition. The more precise characterization and the significance of these subnuclear relocations should be studied in future.

ALS etiology is involved in upper motor neuron from the brain to the spinal cord and lower motor neuron from the spinal cord to muscle. The brain expresses the highest level of the androgen receptor (AR). Androgen-concentrating cells were found to be widely distributed in midbrain, pons, and medulla oblongata, and in the spinal cord, whereas it is less abundant in cranial nerve III, IV, VI, which are spared in ALS^43^,^44^,^45^. The present study suggests AR-mediated signaling pathways could involve in ALS pathogenesis. This might mean a possible role of AR-related pathways in the neurodegeneration of both upper and lower motor neurons. On the other hand, the expansion of a trinucleotide CAG repeat in *AR* gene causes spinal and bulbar muscular atrophy (SBMA), a subtype of motor neuron disease restricted to lower motor neuron involvement, in contrast with ALS characterized by degeneration of both upper and lower motor neurons^46^,^47^,^48^. SBMA affects only males but not females and the pathogenic AR-mediated neurodegeneration is suppressed by androgen inactivation, suggesting the ligand-dependent nuclear accumulation of the polyglutamine (polyQ)-expanded AR protein is toxic especially in lower motor neurons. In contrast to this restricted role of polyQ-expanded AR possibly with loss of AR function in lower motor neurons, the present study could shed a light on a role of AR-mediated pathways in upper motor neurons in ALS pathogenesis.

Epidemiologically the sporadic ALS is characterized by a 2.6:1 higher incidence in men compared with women^49^,^50^,^51^. Various trophic factors are critical in the development and maintenance for nervous system including motor neuron. Spinal cord motor neurons as well as brain express the highest levels of the androgen receptor and androgen is known to have trophic effects on lower motor neurons^44^,^45^. In fact, free testosterone that could cross blood-brain barrier is significantly decreased in ALS patients of both sexes compared with age-matched controls^49^. On the contrary, excessive androgen could be toxic in motor neurons. A low 2D: 4D ratio, a surrogate marker for high prenatal testosterone levels, correlates with a higher risk for ALS^50^. In addition, androgen ablation by orchiectomy resulted in a decrease in androgen receptor levels in spinal cord and skeletal muscle, and this effect correlated with an amelioration of symptoms in human SOD1-G93A mice model^51^. The reduction of serum androgen levels in human SOD1-G93A mice protects from toxicity of mutant protein, whereas excessive androgen stimulation results in deterioration of motor function, aggregation of endogenous androgen receptor in the skeletal muscle and reduced life span. Although the precise role of androgen in motor neurons remains unknown, these findings suggest a possible role of AR-mediated pathways in not only lower motor neurons but also upper motor neurons in ALS etiology.

SAFB1 can interact with and repress transcriptional activity of various nuclear receptors (PPARg, FXRa, RORa1, PPARa, PPARb, VDR, SF1, and LRH-1) other than androgen and estrogen receptor^18^,^19^,^20^. Therefore, the interplay between FUS and SAFB1 could regulate various nuclear hormone receptor-mediated transcriptions in motor neuron.

We also found FUS interacts with Matrin3 along with SAFB1 in Co-IP assay. The mutations in Matrin3 are associated with ALS and distal myopathy^25^. Since Matrin3 also interacts with TDP-43^25^, it is possible the dysfunction of nuclear matrix-associated protein is one of common underlying mechanisms for ALS pathogenesis. Several researchers, including us, reported that over-expression of ALS-linked FUS mutant sequesters various ALS-linked RNA binding proteins such as hnRNP A1/A2 and TDP-43 in cytoplasmic aggregates^35^. Like other ALS-linked RNA-binding proteins, Matrin3 and SAFB1 contain intrinsically disordered domain that can involve in prion-like polymerization to form nonmembranous compartments^52^. We here indicated the cytoplasmic aggregates of ALS-linked mutant FUS P525L sequester Matrin3 and SAFB1 in the aggregation, whereas ectopic FUS P525L apparently lacks the ability of splicing regulation in E1A mini-gene compared with FUS wild type. Although it is still controversial whether ‘loss of nuclear functions’ or ‘gain of toxic functions’ as a result of FUS mutant is critical to trigger ALS, our results suggest these are not mutually exclusive and both could eventually affect the pathogenesis of this devastating disease.

## Materials and Methods

### Yeast two-hybrid Screening

Yeast two-hybrid screening was conducted using Matchmaker GAL4 two-hybrid system 3 (Clontech, CA, USA) on human brain cDNA library as described^26^. The full-length human FUS (amino acid 1-526) was used as a bait.

### Cell Culture

HEK293 (ATCC, CRL-1573 ) and SH-SY5Y(ATCC, CRL-2266) cells were cultured in Dulbecco’s modified Eagle’s medium (DMEM) supplemented with 10% bovine serum at 37 °C in a 5% CO_2_ atmosphere. Transient transfections were performed using Lipofectamine 2000 (Invitrogen, #11668-027).

### Small-interfering RNA (siRNA) Assay

siRNA targeting FUS and SAFB (SAFB 1 and SAFB 2 double-knockdown) were purchased from Greiner Japan. The specific sequences of siRNA for human FUS, or human SAFB were described previously^21^,^53^. A non-targeting control siRNA (Santa Cruz Biotechnologies, sc-37007) was used as a negative control. Transfection of siRNA at 40 nM of final concentration was performed by Lipofectamin 2000 (Invitrogen, #11668-027) according to the manufacture’s protocol. Analysis was performed after 48 h incubation.

### Constructs and mutagenesis

Human cDNAs for *FUS* was provided by the RIKEN BRC through the National Bio-Resource Project of the MEXT Japan (Clone ID #IRAL001I24). cDNAs encoding truncated SAFB1 dN (amino acid 70-915), FUS NT (amino acid 1-270), FUS CT (amino acid 270-526) were generated by PCR. The resulting PCR products were subcloned into pAcGFP1 C1 (Clontech), pGEX (GE Healthcare) or FPC1-Myc or HA^26^ expression vector.

### Western blot analysis, Immunoprecipitation, Immunocytochemistry, and Immunohistochemistry

Experiments involving animals were approved by the institutional Animal Care and Use Committee at Chiba University (Approval number; #A27-12), and the methods were carried out in accordance with the relevant guidelines and regulations. 6-week-old male C57BL6J mice were received from Japan SLC Inc.

Western blot analysis, immunoprecipitation, immunocytochemistry, and immunohistochemistry were performed as described previously^26^. For methanol fixation, cells were fixed in ice-cold 100% methanol for 20 min at −20 °C followed by immunostaining after PBS (−) wash. Pre-fix extraction with detergent (0.5% Triton X-100) was performed as described previously^28^. Briefly, cells were treated with CSK buffer [10 mM PIPES (pH 6.8), 100 mM NaCl, 300 mM sucrose, 3 mM MgCl2, 0.5 mM PMSF] with 0.5% Triton X-100 on ice for 1 min and then fixed with 4% paraformaldehyde for 15 min at room temperature followed by further permeabilization with 0.2% Triton X-100 for 5 min. In regard to the experiments with sonication-mediated chromatin shearing, cell lysates were processed with 3 times sonication for 10 seconds at maximal levels of sonicator (Nissei, TE500). The primary antibodies used were FUS/TLS (Bethyl Laboratories, #A300-302A), FUS/TLS (4H11)(Santa Cruz Bio., sc-47711), SAF-B (F-20) (Santa Cruz Bio., sc-32002), Matrin3 (C-20) (Santa Cruz Bio., sc-55723), Histone H4 (F-9) (Santa Cruz Bio., sc-25260), Histone H1 ( Santa Cruz Bio.,sc-10806), GFP (Santa Cruz Bio., sc-9996), HA (Sigma-Aldrich, HA-7), Actin (I-19) (Santa Cruz Bio., sc-1616), and c-Myc (Santa Cruz Bio., 9E10). In the case of RNase, DNaseI, or Micrococcal nuclease treatment, precipitated immune complexes bound to beads were washed twice with TNE buffer and suspended in each reaction buffer with enzyme (RNase; 0.1 mg/ml RNase A [Takara, # U0505S] for 60 min at 37 °C, RNase-free DNaseI [NEB, #M0303] for 60 min at 37 °C, Micrococcal Nuclease [NEB, #M0247] for 30 min at 37 °C).

Images were obtained using fluorescence microscope (Nikon, E600) equipped with digital camera (DP72, Olympus) or confocal laser scanning microscope (Olympus Fluoview FV10i).

### *In situ* preparation of nuclear matrix for microscopy

*In situ* nuclear matrices were prepared as described^33^. Briefy, cells on coverslips were washed in ice-cold PBS and placed on ice in CSK buffer with 0.5% Triton X-100 for 10 min. Then cells on coverslips were washed with PBS, fixed at room temperature for 15 min with 4% PFA, and incubated on ice for 5 min in extraction buffer [250 mM ammonium sulfate, 300 mM sucrose, 10 mM Pipes (pH 6.8), 3 mM MgCl2, 0.5% Triton X-100, 1 mM PMSF, Complete protease inhibitors (Roche), ribonuclease inhibitor]. Then DNA was digested in digestion buffer [50 mM NaCl, 300 mM sucrose, 10 mM PIPES (pH 6.8), 3 mM MgCl2, 0.5% Triton X-100, 1 mM PMSF, Complete protease inhibitors (Roche), 20 units/mL ribonuclease inhibitor, 500 units/mL RNase-free DNase (NEB, #M0303)] at 37 °C for 1 h. Cells were fixed in 4% PFA, and immunofluorescence was performed. Control samples were stained with DAPI to ensure digestion of DNA.

### Chromatin-bound protein isolation and Chromatin subfraction

The chromatin-bound (CB) protein fraction was obtained as previously described^8^,^9^. Briefly, cells were lysed in a detergent-free lysis buffer (50 mM Tris·HCl, pH 7.4, 150 mM NaCl, 1 mM EDTA) and homogenized by passing through 23G needle 10 times. After centrifugation at 1,000 × g at 4 °C, chromatin and cell debris were in the pellet and non-chromatin-bound proteins were in the supernatant. The pellet was resuspended in the detergent-free lysis buffer and chromatin-bound proteins were released by breaking chromatin DNA with 3 times sonication for 10 s at maximum levels of sonicator (Nissei, #TE500). After centrifugation again at 1,000 × g at 4 °C, chromatin-bound proteins were retained in the supernatant.

S1,S2 and P chromatin subfractions from cultured cell nuclei were obtained as described^30^. Briefly, harvested cells were incubated in hypotonic buffer A [20 mM HEPES pH 7.9, 20 mM NaCl, 5 mM MgCl_2_, 1 mM ATP] on ice for 15 min. Lysis of the cytoplasmic membrane was conducted with 20 strokes in a 15 ml cell douncer. The nuclear pellet, obtained by centrifugation, was resuspended in buffer B [20 mM HEPES pH 7.9, 0.15 M NaCl, 0.5 mM MgCl_2_, 0.3 mM sucrose, 2 mM CaCl_2_, 1 mM ATP, and 0.5% NP-40] for 30 min at 4 °C and then centrifuged to obtain a total chromatin fraction and a soluble nuclear fraction. The chromatin fraction, resuspended in buffer B without NP-40, was incubated for 4 min at 16 °C with 30 U (1 μl) of Micrococcal nuclease (NEB, #M0247). The solution was centrifuged to obtain the supernatant (S1). The pellet was resuspended in 8 mM EDTA, incubated at 4 °C for 15 min, and centrifugated to obtain the chromatin fraction (P) and supernatant (S2). The proteins contained in each fraction were separated by 12.5% SDS-PAGE and subjected to Western blot.

### Pull down assay

Pull down assay was performed as described previously^54^. GST fusion proteins were expressed in BL21 *Escherichia coli* cells, and crude bacterial lysates were prepared by sonication in GST lysis buffer (25 mM Tris at pH 7.5, 150 mM NaCl, 1 mM EDTA, protease inhibitor). The lysates were added with 30 μl of glutathione-Sepharose beads (50% slurry) and mixed for another 1 h at 4 °C. The beads were then washed three times with the above GST lysis buffer. Approximately 10 μg of each GST fusion protein was incubated with 100 μg cell extract for 1 h at 4 °C. After the beads were washed three times in GST lysis buffer, proteins from the beads were separated on 10% SDS-PAGE for Western blot.

### Luciferase activity assay

In luciferase assays, HEK293 cells were cultured in DMEM with 10% charcoal stripped fetal bovine serum (Biological Industries, Cat#04-201-1A). pOLuc-1630 Luc plasmid was kindly provided by Dr. Peter Rotwein (Oregon Health and Science University)^38^. Cells were plated at 5 × 10^4^ cells per well onto wells in 24-well plate. On the next day, cells were transfected with various plasmids including 0.25 μg pEGFP-C1-AR (Addgene, #28235), 0.25 μg pOc1630 Luc, and 0.25 μg pSV-β-Galactosidase Vector (Promega, #E1081) per well using Lipofectamine 2000 (Invitrogen, #11668-027)). Total plasmid amount was equalized for each transfection using the appropriate empty vector. At 12 h after transfection, cells were treated with mock or 80 nM 5a-dihydrotestosterone (TCI, #A0462). After 24 h incubation, firefly luciferase activities in cell lysates were measured using Luciferase Reporter Assay Kit (BioVision, #K801-200) with Berthold MicroLumat Plus LB96V. Data were normalized by the β-Gal activities using pSV-β-Galactosidase Vector. The luciferase activity relative to the untreated control was determined, and results are presented as means (± SD) of triplicates.

### *In vivo* splicing Assay

*In vivo* splicing assays were performed as described previously^34^,^55^,^56^. E1A expression vector (pCEP4‐E1A) was kindly provided by Dr. Tarn WY (Institute of Biomedical Sciences, Taipei, Taiwan). Briefly, HEK293 cells were co-transfected with pCEP4-E1A and expression plasmid encoding effector protein, including FUS or SAFB1, in a 35 mm dish. Total plasmid amount was equalized for each transfection using the appropriate empty vector. At 48 h pos-transfection, total RNA was isolated using TRIzol (Invitrogen), and subjected to RT (reverse transcription) after DNase I treatment with ReverTra Ace^®^ qPCR RT Master Mix with gDNA Remover (TOYOBO, # FSQ-301). RT was performed with primer P3 (5′-CGGTATTCCACATTTGGACACT-3′). Semi-quantitative PCR was performed with primers P1 (5′-GGTCTTGCAGGCTCCGGTTCTGGC-3′) and P2 (5′-GCAAGCTTGAGTGCCAGCGAGTAG-3′) in GeneAmp PCR System 9700 (Applied Biosystems). The total number of cycles in PCR program was optimized to reach the linear phase. PCR products were visualized on 5% polyacrylamide gel electrophoresis with 6× DNA fluorescent loading dye (SMOBIO TECHNOLOGY, #DL1000). A DNA ladder molecular weight marker (Bionexus, #BN2050) was run on every gel to confirm expected molecular weight of the amplification product. The percentage of each transcript signal relative to the total amount of the five splicing variants (9S, 10S, 11S, 12S, 13S) was calculated for each sample and expressed relative to the appropriate control splice form percentages, which were set equal to 1 as described^56^.

### Quantification for Western Blot and PCR Product Bands

Western blot and PCR product images were analyzed with ImageJ (National Institutes of Health), which evaluates the relative amount of protein or DNA staining with normalization to the corresponding controls.

### Statistical Analysis

Data were expressed as means ± standard deviation (SD). Quantitative data were analyzed by the Student’s *t*-test for two groups or analysis of variance (ANOVA) with Bonferroni post-hoc statistical analysis for three or more group comparisons using Statcel (Add-in software for Microsoft Excel, OMS Ltd., Japan). *p-*values less than 0.05 were considered statistically significant. Quantification of at least three replicates for each condition was shown.

## Additional Information

**How to cite this article**: Yamaguchi, A. and Takanashi, K. FUS interacts with nuclear matrix-associated protein SAFB1 as well as Matrin3 to regulate splicing and ligand-mediated transcription. *Sci. Rep.* **6**, 35195; doi: 10.1038/srep35195 (2016).

## Supplementary Material

Supplementary Information
