# Supplementary material for: FUS interacts with nuclear matrix-associated protein SAFB1 as well as Matrin3 to regulate splicing and ligand-mediated transcription
Source: Sci Rep. 2016 Oct 12;6:35195. doi: 10.1038/srep35195 (PMC5059712; doi:10.1038/srep35195)

**FUS interacts with nuclear matrix-associated protein SAFB1 as well as Matrin3 to regulate splicing and ligand-mediated transcription**

**Atsushi Yamaguchi^1^, Keisuke Takanashi**^1^**,**

^1^Department of Neurobiology, Graduate School of Medicine, Chiba University, Chiba, Japan

**Supplementary Figure 1.**

(A) SH-SY5Y cells were fixed with 4 % paraformaldehyde, permeabilized with 0.2 % TritonX-100, and immunostained with anti-FUS monoclonal (#sc-4711) or polyclonal (#A300-302A) antibody followed by fluorescent secondary antibody. Images were obtained with fluorescence microscope after DAPI staining. Scale bar: 2 μm.

(B) SH-SY5Y cells were fixed with 4 % paraformaldehyde and permeabilized with 0.2 % TritonX-100 (left panels) or treated with CSK buffer + 0.5% TritonX-100 as described in ‘Materials and Methods’ (middle panels) or fixed with ice-cold methanol for 20 min at -20 ºC (right panels). Then cells were stained with anti-FUS monoclonal (#sc-4711) antibody followed by fluorescent secondary antibody. Images were obtained with fluorescence microscope after DAPI staining. Scale bar: 2 μm.

**Supplementary Figure 2.**

(A) Co-IP assay between FUS and SAFB1 with RNase treatment. Lysates of HEK293 with sonication-sheared chromatins were immunoprecipitated with mouse IgG (control) or anti-FUS antibody in the presence or absence of RNase A treatment (RNase + or -), and followed by the immunoblot with anti-SAFB1 antibody (upper panel). The membrane was reprobed with anti-FUS antibody (lower panel).

(B)(C) Effects of siRNA-mediated knockdown of FUS or SAFB protein levels. HEK293 cells were transfected with 40nM FUS- or SAFB-specific siRNA. At 48 h after transfection, cells were lysed for Western blot with anti-FUS or anti-SAFB antibody. Membranes were reprobed with anti-Actin antibody as loading control (lower panels). The relative ratio of SAFB (B) or FUS (C) protein between specific siRNA and control siRNA was shown in lower graph. The data are presented as the mean values ± SD that were repeated a total of three times.

(D) Schematic diagrams showing structures of deletion mutant FUS-NT and FUS-CT. Bacterially produced GST-FUS wt, GST-FUS-NT, or GST-FUS-CT (10 μg each) was incubated with 200 μg lysates of HEK293 over-expressing Myc-SAFB1, and subjected to Western blot with anti-SAFB1 antibody. The lower panel shows the CBB-stained SDS-polyacrylamide gel with GST-fused proteins used in the pull down experiments.

**Supplementary Figure 3.**

*In situ* nuclear matrices in SH-SY5Y were prepared as described in ‘Materials and Methods’. SH-SY5Y cells were co-stained with anti-FUS (#A300-302A) and anti-SC35 antibody. Upper panels (Cont) are control images fixed with 4 % PFA and permeabilized with 0.2 % Triton-X 100. Lower panels (In situ NM) are images of *in situ* nuclear matrices. Images were obtained with fluorescence microscope after DAPI staining.

**Supplementary Figure 4.**

(A) HEK293 cells were transfected with HA-FUS wt or P525L plasmid for 48 h. Then cells were fixed (4 % PFA), permeabilized (0.2 % TritonX), and stained with anti-HA antibody followed by fluorescent secondary antibody. Images were obtained with fluorescence microscope after DAPI staining. Scale bar: 2 μm.

(B) HEK293 cells were transfected with HA-FUS wt or P525L plasmid for 48 h. Then chromatin subfractions (S1, S2, P) were prepared, and subjected to Western blot with anti-SAFB1 (upper), -HA (middle), and -Histone H1 (bottom) antibody. The relative ratio of P to S1 fraction of HA-FUS (left graph) and SAFB1 (right graph) were shown in lower graphs. The data are presented as the mean values ± SD that were repeated a total of three times. *** *p*** < 0.05 with Student’s ***t***-test.


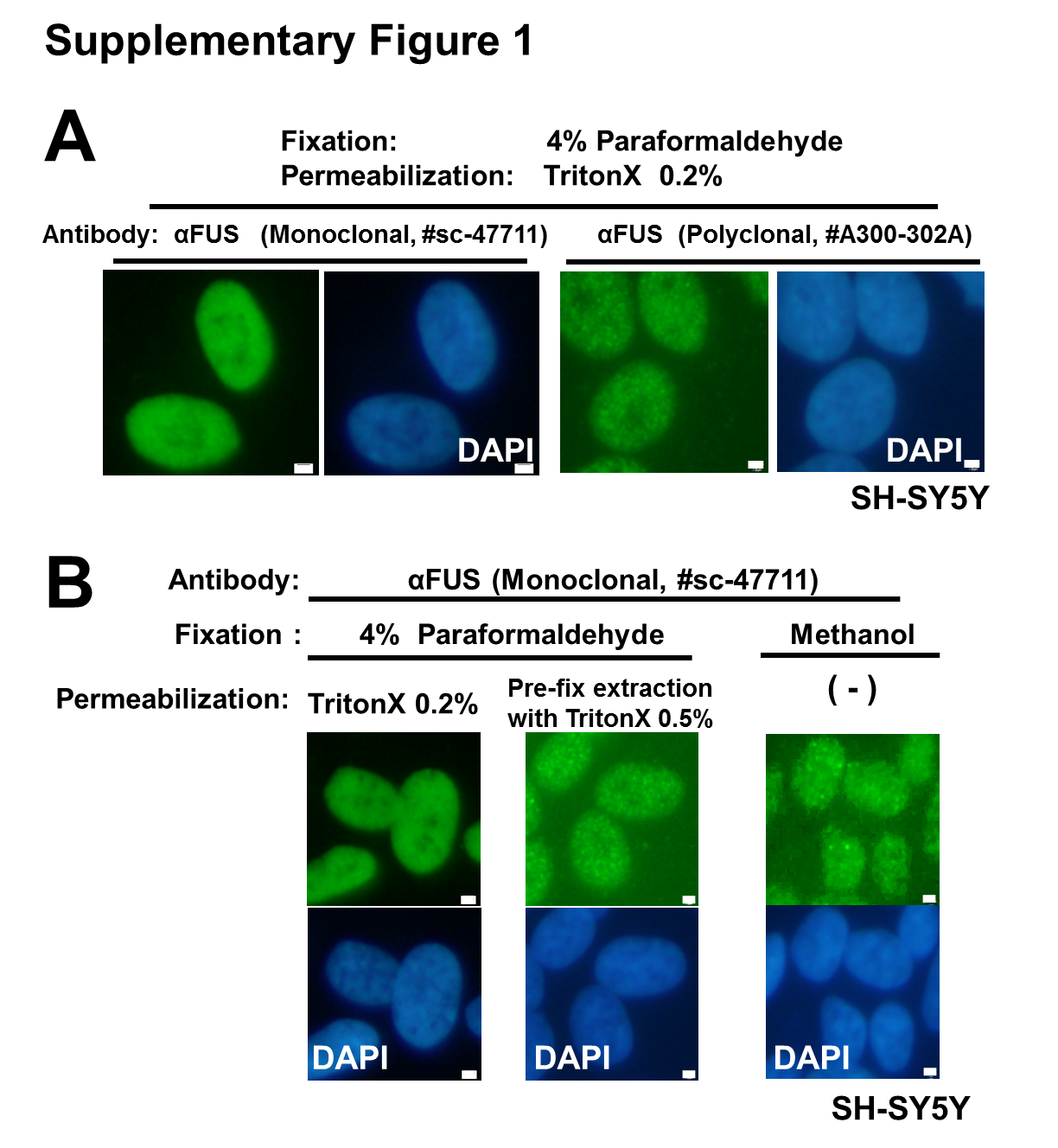


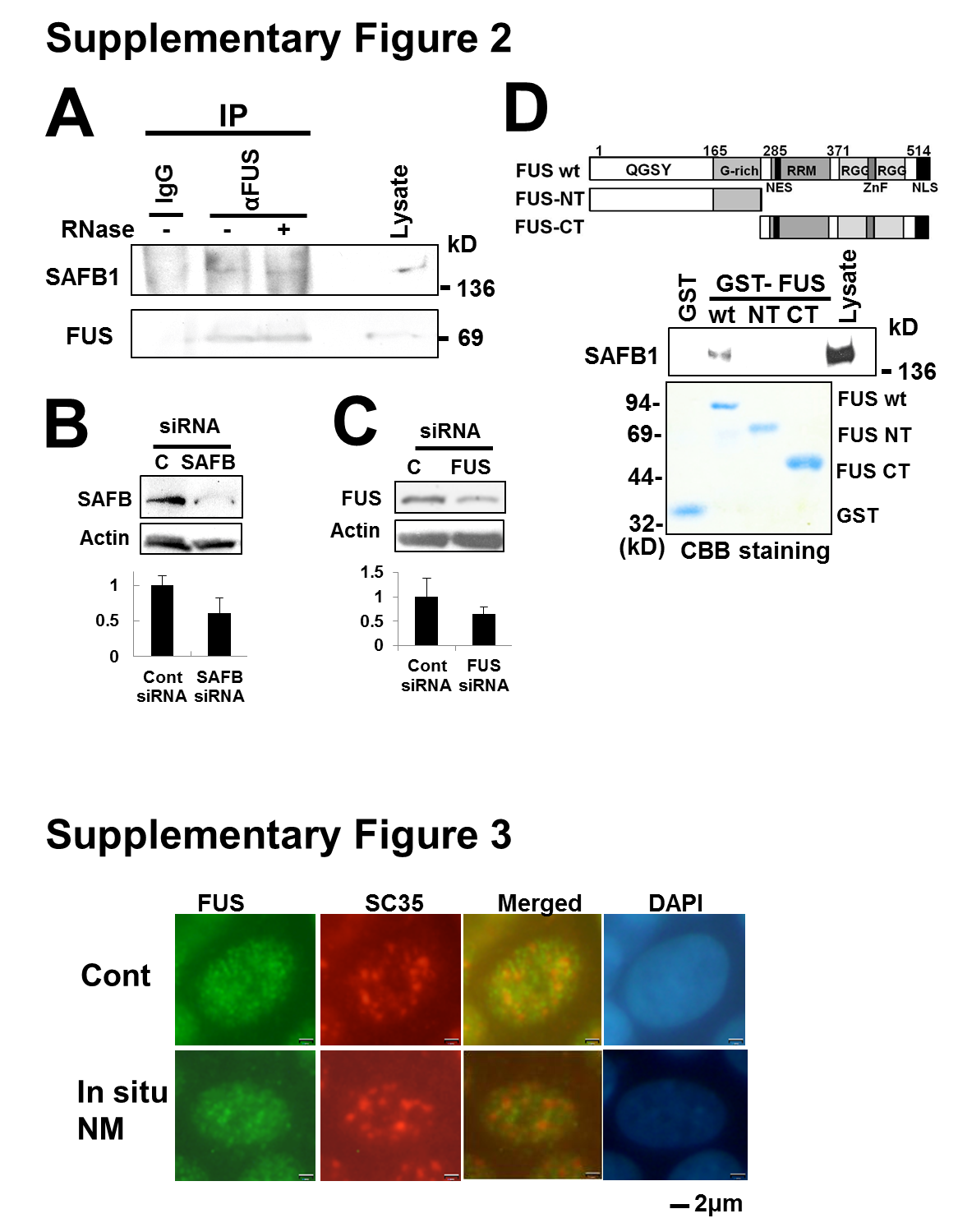


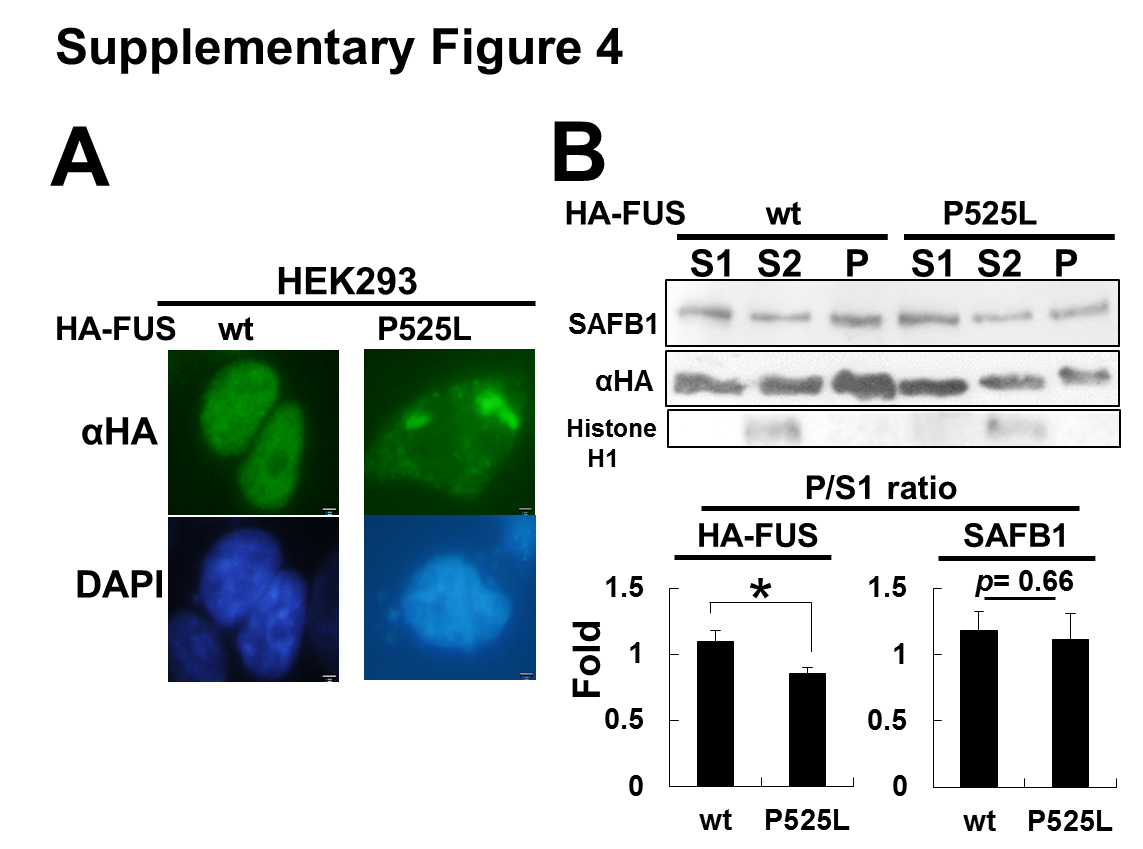

Supplement: Supplementary Information [file srep35195-s1.doc]
